# A Prospective Evaluation of Grip Strength Comparing a Low-Tech Method to Dynanometry in Preoperative Surgical Patients and Weak Intensive Care Patients

**DOI:** 10.1155/2022/3428851

**Published:** 2022-10-19

**Authors:** Mark J. Shea, Anika Weightman, Bradley Wibrow, Matthew H. Anstey

**Affiliations:** ^1^Armadale Hospital, Mount Nasura, Western Australia, Australia; ^2^Sir Charles Gairdner Hospital, Nedlands, Western Australia, Australia; ^3^University of Western Australia, Perth, Australia; ^4^Curtin University, Perth, Australia

## Abstract

**Objective:**

Grip strength testing offers a mechanism to identify patients in whom frailty might be present, discriminate between robust elderly and vulnerable younger patients, and can be used as a tool to track changes in muscle bulk over the course of an inpatient stay. We compared gold-standard quantitative grip strength measurement to a low-tech alternative, a manual bedside sphygmomanometer.

**Design:**

Under supervision, subjects performed hand-grip strength testing with each instrument. A mean score is calculated from three measurements on the dominant and nondominant hand. *Setting*. Testing was performed in a tertiary centre in Perth, Western Australia, in both outpatient clinics and intensive care units. *Participants*. 51 adult pre-operative surgical outpatients were assessed, alongside 20 intensive care inpatients identified as being weak. *Main outcome measures*. A statistical correlation between the two measures was evaluated. Feasibility, safety, and convenience were also assessed in outpatient and bedside settings.

**Results:**

Highly correlated results in both tertiary surgical outpatients (*r*_s_ = 0.895, *p* ≤ 0.001, *N* = 102; *r* (100) = 0.899, *p* ≤ 0.001) and weak intensive care patients (*r*_s_ = 0.933, *p* ≤ 0.001, *N* = 39 r (37) = 0.935, *p* ≤ 0.001)

**Conclusions:**

Modifying a manual bedside sphygmomanometer to measure grip strength is feasible and correlates well with a formal dynamometer in preadmission surgical patients and weak patients in the intensive care unit. The use of an existing, safe, and available device removes barriers to the measurement of weakness in patients and may encourage uptake of objective measurement in multiple settings.

## 1. Introduction

Frailty is defined as “decline in multiple body systems, which increases an individual's vulnerability to changes in health or their environment” [1]. Grip strength may be used alone to screen for frailty [2–4], or as part of a frailty scoring system [5]. Measuring grip strength is simple and rapid, though not routinely used in clinical practice outside of research settings [6]. Scoring systems that attempt to evaluate frailty without a functional test have been shown to not be predictive of outcome [7], whereas those that include a functional test have been shown to predict mortality in general [8], trauma [7], and surgical patients [9–12]. Separately, grip strength alone may predict extubation success [13], identify intensive care unit acquired weakness (ICUAW) [14–16], and predict outcomes in ventilated chronic obstructive pulmonary disease (COPD) patients [17]. In a more diverse French intensive care population, grip strength was shown to be predictive of difficult weaning from ventilation [18].

Barriers to the evaluation of grip strength include the limited availability of a dynamometer, in part due to its being relatively expensive, fragile, and requiring specific training. Its use may also be limited due to infection control precautions. In contrast, the manual sphygmomanometer is widely available and prior research in healthy volunteers established that it accurately measured grip strength [19,20].

Grip strength testing offers a noninvasive mechanism to identify patients in whom frailty might be occult, where a more extensive frailty index is unwieldy, impractical, time-consuming, or where clinical suspicion might need to be confirmed [21–23]. It can also be used as a tool to track recovery or deterioration in muscle strength in the intensive care unit (ICU) [24]. Early frailty [21] and frailty in the obese [25] are challenging to identify; objective functional testing offers a way to improve sensitivity in screening. Gait-speed, six-minute-walk test results, and timed “get up and go” tests remain alternatives to quantitative grip strength measurement that do not require specialist equipment [25], but are comparatively lengthy and not useful in a group of people who have difficulty walking or are critically unwell. The complete frailty indices available take considerable time to carry out, and simple screening tests are attractive for busy clinicians [12]. Brief scoring systems and the more complete Fried frailty assessment both require assessment of grip strength. Existing systems that ask clinicians to rate patients' frailty on a scale–for example, the Canadian Frailty Scale (CFS)—have advantages over “end of the bed” assessment [26] but have deficits compared to evaluations that include objective outpatient testing [21].

Given the utility of these measurements and the weaknesses of the dynamometer, we chose to make a comparison of the dynamometer's measurements to those of a manual sphygmomanometer. The use of a more easily available device would allow easier grip strength testing and facilitate the addition of objective, functional information. Showing the correlation of these measurements might enable further research in premorbid patients, patients within the ICU, and in recovered patients. For these reasons, and to further validate the apparatus, we selected an outpatient group as well as a weak ICU cohort.

## 2. Materials and Methods

Patients were enrolled at Sir Charles Gairdner Hospital, a tertiary level teaching hospital in Western Australia. Two cohorts were selected: a convenience sample of elective surgical patients in a preadmission clinic; and intensive care patients identified as weak by the treating intensivists.

Patients were required to be competent to consent and obey commands sufficiently to engage in measurement. Patients were recruited between September 2018 and March 2019. Approval was granted as a quality improvement project (GEKO ^#^26469).

We used the Jamar plus hand dynamometer (Sammons Preston, Bolingbrook, IL) and compared it to a manual aneroid sphygmomanometer (ABN Healthcare Systems).

**Figure 1:**
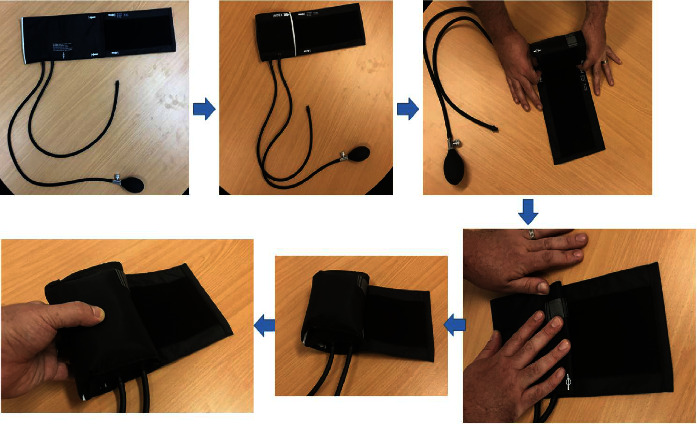
Creation of the blood pressure cuff for grip strength testing. The sphygmomanometer starts fully open, then, rolled closed to its minimum size on the fastening surface, then, inflated to 20 mm·Hg, resulting in a hand-sized pillow.

Figure 1 illustrates how to create the blood pressure cuff into a grip strength tool. To use the sphygmomanometer, the cuff is opened fully, and then, folded as if to bring it to its “smallest” size [19]. The result is a small pillow. The cuff is then inflated until the pressure gauge reads 20 mm·Hg; a moment is taken to allow the air to distribute through the internal bladder fully, and then, the measurement is checked again; the cuff may also be inflated above this threshold for a period, then, deflated back to 20 mm·Hg.

All patients were assessed with both the Jamar dynamometer and the manual sphygmomanometer as originally described by Fess and Moran [27]. Patients sat with shoulder adducted, elbow flexed to 90 degrees, wrist 0–30 degrees dorsiflexion. Both upper limbs were assessed with both measurement devices. Three successive trials were made with vocal encouragement [19, 20, 27]; the mean of three trials was recorded as the grip strength for each limb. The measurement of peak exerted force was recorded for each trial made with each hand, with both the dynamometer and the sphygmomanometer read off the pressure gauge.

Twenty patients in the intensive care unit were also evaluated. These patients were chosen if they were felt to be globally weak by the treating intensivist. Patients were excluded if they could not give consent, could not obey commands, could not be positioned so as to adequately perform the measurements, or had suffered direct injury or pathology to either upper limb (for example, pressure injury or local infection).

Demographic data were collected from both groups of patients.

## 3. Results

**Figure 2:**
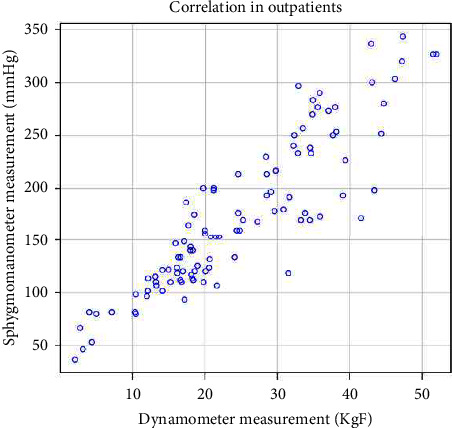
Correlation between grip strength techniques in preadmission surgical patients.

Fifty-one participants from the preadmission clinic completed the testing procedure. These are summarised in Table 1.  Figure 2 plots the grip strength using the two techniques (*n* = 102, 51 patients).

The Spearman's rho correlation coefficient was used to assess the relationship between the two grip strength measures in outpatients; there was a significant correlation between the two, *r*_s_ = 0.895, *p* ≤ 0.001, *N* = 102. Also, Pearson's product-moment correlation was used, also finding a significant correlation r(100) = 0.899, *p* ≤ 0.001.

**Figure 3:**
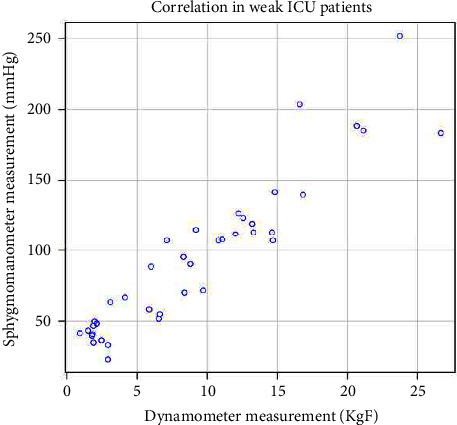
Correlation between grip strength techniques in weak ICU patients.

A linear regression model was used to predict a dynamometer measurement (KgF) from a sphygmomanometer measurement (mm·Hg); *R*^2^ = 0.96, *R*^2^_adjusted_ = 0.96; the regression coefficient (*B* = 0.14, 95%CI [0.14–0.15]) indicated that an increase in sphygmomanometer measurement by 1 mm·Hg corresponded to an increase in dynamometer measurement of 0.14 KgF.  Figure 3 outlines the correlation in the weak ICU patients (*n* = 39, 20 patients).

The Spearman's rho correlation coefficient was used to assess the relationship between the two grip strength measures in weak ICU patients; there was a significant correlation between the two, *r*_s_ = 0.933, *p* ≤ 0.001, *N* = 39. Pearson's product-moment correlation also found a significant correlation r (37) = 0.935, *p* ≤ 0.001.

The linear regression model (mm·Hg); *R*^2^ = 0.87, R^2^_adjusted_ = 0.87; the regression coefficient (*B* = 0.11, 95% CI [0.10–0.13]) indicated that an increase in sphygmomanometer measurement by 1 mm·Hg corresponded to an increase in dynamometer measurement of 0.11 KgF. Supplementary Table 1 provides a description of the characteristics of the ICU patients who were “designated weak.”

Feasibility of testing: no patients found the experience uncomfortable or difficult. The testing took less than ten minutes, and in the intensive care setting, this procedure was easy to perform between episodes of care and intensive care nurses reported it to be easy to do and had no significant impact on nursing workload.

## 4. Discussion

The use of a low-tech measure of grip strength performed very well compared to the use of a standard dynamometer. The correlation between the measurements made with the sphygmomanometer and those made with the dynamometer was very high in this population. Previous work has shown these measures to be largely comparable, albeit in a population of well, younger, stronger females [19] and healthy volunteers [20]. The Jamar device is taken as a gold standard, although there are competitor devices that have been evaluated in the published literature, such as the Rolyan [28] and Bodygrip [29] dynamometers.

The use of the low-tech measure of grip strength also performed well in weak intensive-care patients; the correlation remained high (0.935). The device was noninvasive, useable, and safe in the intensive care unit. Normative data available for the Jamar device in Australia [30] and Canadian research [5] describes a weak relationship to BMI but a clear difference in sex; patients are said to be abnormally weak if they fall into the weakest 20% of their sex and BMI cohort. Dynamometry readings of <29 kgF in males and <17 kgF in females meet the cutoff for the grip strength component (dominant hand) of frailty scoring and <11 kgF in males, <7 kgF in females is the definition used for ICUAW [14, 24]. In the sampled outpatients, 20 of 51 (8 females and 12 males) met the Fried cutoff; in the sampled weak ICU patients, 9 of 20 (5 females and 4 males) met the criteria for ICUAW, with all ICU patients recording grip strengths under the Fried limit. Based on linear regression in the outpatient dataset, the predicted strength in KgF is 0.14*∗*(strength in mmHg). These values reflect approximately 50–210 mm·Hg, an area easy to evaluate on the gauge.

A low-tech, more readily available measure of grip strength might increase screening for ICU-acquired weakness. Quantitative grip strength assessment has been shown to be sensitive in detecting ICUAW [24]. The sphygmomanometer is familiar, safe, and often disposable or single-use, which is of benefit where there are infection control precautions. A high standard of calibration also exists for the device. For this reason, the sphygmomanometer would be an excellent substitute for the dynamometer in many clinical scenarios. The familiarity, ubiquity, and availability of the sphygmomanometer would allow quantitative grip strength to be incorporated into ICU nursing assessment or ICU physician assessment inside and outside the ICU.

Conceptually, there is overlap between the concepts of frailty and acquired weakness. While frailty is a prehospital diagnosis, weak patients assessed in the ICU might be frail or might have developed weakness after admission. Grip strength assessment therefore might be helpful in identifying patients who will be at need of significant rehabilitation, or prioritise for early referral to specialist rehabilitation [31].

There are several limitations to this study. It was more difficult to recruit the weak group in intensive care than anticipated; the principal barrier was identification of weak ICU patients who also had appropriate capacity to consent to research and obey commands. This group was chosen to demonstrate that the device could still reproduce lower figures in a group expected to have lower grip strength, which it did. The use of a convenience sample for the outpatient group is a potential problem. However, this comparison has not previously been made in any patient population of which we are aware, and we did not expect significant biases using a convenience sample evaluating this technique.

The sphygmomanometer is likely to underestimate the strength in very strong patients due to its upper limit of accurate measurement at approximately 300 mm·Hg-although suitable for blood pressure measurement, this might be exceeded by very robust patients. For this reason, we cannot assess the validity of the comparison outside this range of strength.

Grip strength is, by convention, evaluated in the distal upper limb in a standardised position–while this is the published standard, this may not reflect the degree of whole-body sarcopenia expected in frailty. This is of interest in the ICU, where the syndrome of intensive care mononeuropathy tends to preserve distal power relative to proximal limb power and truncal stability; patients may thus “overperform” if their distal strength is measured, and appear less weak than they are. Finally, we did not evaluate this measurement chronologically in ICU patients; it might be desirable to evaluate how this measure changes over time in a subsequent study.

## 5. Conclusions

Modifying a manual bedside sphygmomanometer to measure grip strength is feasible and correlates well with a formal dynamometer in preadmission surgical patients and weak patients in the intensive care unit. The use of this commonly available device enables the measurement of grip strength objectively, a result that has utility for the identification of patients with acquired weakness in the hospitals, quantitative evaluation of progress after intensive care, and improved sensitivity in the identification of frail patients. The use of an existing, safe, and available device removes barriers to the measurement of weakness in patients and may encourage the uptake of objective measurement in multiple settings.

## Figures and Tables

**Table 1:** Descriptive data for both cohorts.

		Preadmission clinic patients	Weak intensive care patients
Total patients (*n*)		51	20
Male gender (%)		29 (57%)	9 (45%)
*Age (years)*	Mean	62.8	67.75
	Median	65	68
	Q1-Q3 (IQR)	55.5–73.5 (18)	59.25–73.25 (14)
Weight (kg)	Mean	85.12	73.28
	Median	80	63.95
	Q1-Q3 (IQR)	68.50–94 (25.5)	55.30–86.12 (30.82)
Grip Strength (kg F)	Mean	24.668	9.25
	Median	21.3	8.37
	Q1-Q3 (IQR)	16.67–33.75 (17.08)	2.93–13.22 (10.28)
Grip Strength (mm·Hg)	Mean	172.91	94.68
	Median	160	90
	Q1-Q3 (IQR)	117–224 (107)	49.17–117.17 (68)
Spearman's rank correlation	*n*	120	39
	rho	0.895	0.933
	*p*	<0.001	<0.001
Pearson's product moment correlation	df	100	37
	rho	0.899	0.935
	*p*	<0.001	<0.001

## Data Availability

The data used to support the findings of this study are available from the corresponding author upon reasonable request.

## Supplementary Materials

Supplementary Materials
*Table 1*. Description of weak ICU patients.
