# Supplementary material for: A Prospective Evaluation of Grip Strength Comparing a Low-Tech Method to Dynanometry in Preoperative Surgical Patients and Weak Intensive Care Patients
Source: Crit Care Res Pract. 2022 Oct 19;2022:3428851. doi: 10.1155/2022/3428851 (PMC9605854; doi:10.1155/2022/3428851)
Supplement: Supplementary Materials — Table 1. Description of weak ICU patients. [file 3428851.f1.docx]

Supplementary Table 1. Description of weak ICU patients.
